# Management of ventilator-associated pneumonia in intensive care units: a mixed methods study assessing barriers and facilitators to guideline adherence

**DOI:** 10.1186/s12879-016-1665-1

**Published:** 2016-07-22

**Authors:** Nasia Safdar, Jackson S Musuuza, Anping Xie, Ann Schoofs Hundt, Matthew Hall, Kenneth Wood, Pascale Carayon

**Affiliations:** William S. Middleton Memorial Veterans Hospital, Madison, WI USA; Department of Medicine, University of Wisconsin School of Medicine and Public Health, Madison, WI USA; Department of Infectious Disease, University of Wisconsin Hospital and Clinics, Madison, WI USA; Institute for Clinical and Translational Research, University of Wisconsin, Madison, WI USA; Armstrong Institute for Patient Safety and Quality, Johns Hopkins University School of Medicine, Baltimore, MD USA; Center for Quality and Productivity Improvement, University of Wisconsin-Madison, Madison, WI USA; Department of Infectious Medicine, Marshfield Clinic, Marshfield, WI USA; R Adams Cowley Shock Trauma Center, University of Maryland School of Medicine, Baltimore, MD USA

**Keywords:** Ventilator-associated pneumonia, VAP, Guidelines, Systems Engineering Initiative for Patient Safety, Barriers and facilitators

## Abstract

**Background:**

Guidelines from the Infectious Diseases Society of America/The American Thoracic Society (IDSA/ATS) provide recommendations for diagnosis and treatment of ventilator-associated pneumonia (VAP). However, the mere presence of guidelines is rarely sufficient to promote widespread adoption and uptake. Using the Systems Engineering Initiative for Patient Safety (SEIPS) model framework, we undertook a study to understand barriers and facilitators to the adoption of the IDSA/ATS guidelines.

**Methods:**

We conducted surveys and focus group discussions of different health care providers involved in the management of VAP. The setting was medical-surgical ICUs at a tertiary academic hospital and a large multispecialty rural hospital in Wisconsin, USA.

**Results:**

Overall, we found that 55 % of participants indicated that they were aware of the IDSA/ATS guideline. The top ranked barriers to VAP management included: 1) having multiple physician groups managing VAP, 2) variation in VAP management by differing ICU services, 3) physicians and level of training, and 4) renal failure complicating doses of antibiotics.

Facilitators to VAP management included presence of multidisciplinary rounds that include nurses, pharmacist and respiratory therapists, and awareness of the IDSA/ATS guideline. This awareness was associated with receiving effective training on management of VAP, keeping up to date on nosocomial infection literature, and belief that performing a bronchoscopy to diagnose VAP would help with expeditious diagnosis of VAP.

**Conclusions:**

Findings from our study complement existing studies by identifying perceptions of the many different types of healthcare workers in ICU settings. These findings have implications for antibiotic stewardship teams, clinicians, and organizational leaders.

## Background

Ventilator-associated pneumonia (VAP) is the most common nosocomial infection in the intensive care unit (ICU), with an incidence ranging from 9 % to as high as 39 % [1–4]. VAP is associated with prolonged hospitalization, health care costs and high mortality rates [5–7]. Healthcare-associated pneumonia, the majority of which in the ICU is VAP, accounts for more than 50 % of the antibiotics prescribed in the ICU and a significant proportion of inappropriate and overprescribed antibiotics [8, 9]. Several studies have shown that inappropriate treatment of VAP leads to adverse outcomes [10, 11].

Guidelines from the Infectious Diseases Society of America/The American Thoracic Society (IDSA/ATS) provide recommendations for diagnosis and treatment of VAP [12]. However, it has become clear that the mere presence of a guideline, while necessary, is rarely sufficient to promote widespread adoption and uptake [13–23]. It is unclear as to what extent these guidelines are currently followed in ICUs and what barriers may exist that impede the implementation of these guidelines. A growing body of literature has identified barriers that must be addressed for guidelines to be effectively incorporated into clinical practice [24, 25]. We used a novel systems engineering framework (Systems Engineering Initiative for Patient Safety-SEIPS) to develop and administer a survey to understand barriers and facilitators to the adoption of the IDSA/ATS guideline.

These barriers may be broadly categorized into the five elements of the work system component of the SEIPS model [26]: 1) characteristics of the guideline to be introduced (the ‘tool/technology’ element of the work system model), 2) characteristics of the individuals who play a role in adoption and usage of the guideline (the ‘individual’ element), 3) use of the guideline for changing behavior (the ‘task’ element), 4) characteristics of the organization in which the change is to occur, such as extent of training (the ‘organization’ element), and 5) characteristics of the physical environment in which the change is to occur (the ‘environment’ element) [27]. The objective of this study was to characterize the barriers and facilitators of guideline-concordant care for VAP management.

## Methods

### Settings

This study was conducted in medical-surgical ICUs at the University of Wisconsin Hospital, a tertiary academic hospital (hospital A) and St. Joseph’s Hospital, a large multispecialty rural hospital (hospital B) in the Midwestern US. The medical-surgical ICU at the University of Wisconsin is a 24 bed ICU staffed by board certified intensivists and anesthesiologists. The medical-surgical ICU at St Joseph’s hospital is also staffed by trained intensivists. The study was conducted in 2008–2009.

### Data collection

#### Focus group methods and development of survey

**Fig. 1:**
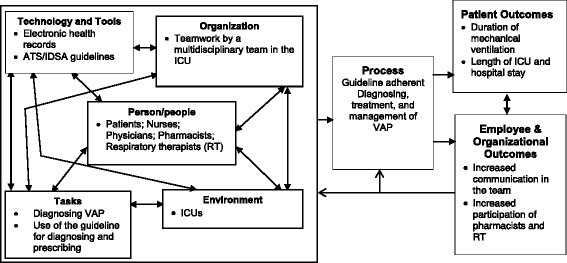
Adaptation of the SEIPS model to the Management of Ventilator-Associated Pneumonia in Intensive Care Units. The five interacting components of the work system part of the SEIPS model are shown, the process involved and the resulting outcomes

To identify barriers and facilitators of managing VAP in ICUs from the providers’ perspective, a survey was developed based on literature review and content analysis of data collected from focus groups. Two focus group discussions (FGDs) (one with physicians and one with nurses, respiratory therapists and pharmacists) were conducted at each of the two hospitals. The objective of the FGDs was to identify and discuss issues of VAP management using the SEIPS framework (Fig. 1). Content analysis of focus groups’ transcripts identified a total of 193 “unique” comments, which were further classified into 9 themes: (1) communication between providers, (2) difficulty in diagnosing VAP, (3) education related to VAP and VAP management, (4) guideline awareness and use, (5) management of the condition, (6) provider responsibilities, (7) technology and its use, (8) use of clinically indicated tests, and (9) variation in practice. Based on the comments and themes identified, a survey was developed and pilot tested on four participants, all of whom were involved in medical management of VAP. They included one physician, one nurse, one respiratory therapist and one pharmacist. Revisions to the content of the questions were made to the survey according to the feedback from pilot testing.

The final survey was adapted to the two hospitals. The version developed for hospital A consisted of five sections. Background had two questions asking the participants if they were aware of the IDSA/ATS guideline for VAP management and to what extent they were willing to follow the guideline. Methods asked participants to indicate to what extent they agree or disagree with 45 statements on VAP management, using a 5-point Likert scale (1 strongly agree, 2 agree, 3 neither agree nor disagree, 4 disagree and 5 strongly disagree). Results asked participants to indicate the frequency with which 17 situations occurred in VAP management. This used a 5-point Likert scale (1 rarely, 2 occasionally, 3 sometimes, 4 fairly often and 5 very often). Discussion had five yes/no questions. These addressed VAP management and diagnosis guidelines and assessed considerations regarding the mini-bronchoalveolar lavage (mini-BAL) procedure. Conclusion collected demographic information of participants including gender, age, job position, work shift, and average length working for the present employer in the current position.

The survey developed for hospital B similarly had five sections, but a few questions were different from those for hospital A. This was because, in addition to the IDSA/ATS guideline for management of VAP, hospital A also has its own VAP management guidelines that were largely similar to national guidelines but not identical.

#### Procedure

The paper survey was distributed to all physicians, nurses, respiratory therapists and pharmacists who practiced in the medical-surgical ICUs at both hospitals. The study was approved by the University of Wisconsin-Madison and the Marshfield Clinic Institutional Review Boards (IRBs). We obtained informed consent from all participants involved in the study.

#### Analysis

Survey data was entered (with verification for accuracy) in an SPSS database. Descriptive statistics were calculated to identify top-ranked barriers and facilitators of VAP management. For each question, the number and percentage of participants who chose each of the available response categories were calculated. If the wording of a question was positive (e.g., pharmacist participation on ICU rounds is beneficial), then participants who chose “agree” and “strongly agree” or “fairly often” and “very often,” (when the question was about frequency), considered it as a facilitator; and participants who chose “disagree” and “strongly disagree” or “occasionally” and “rarely” considered it as a barrier. In contrast, if a question was stated in a negative manner (e.g., having multiple groups of physicians manage patients in the ICU complicates VAP guideline use), then participants who chose “agree” and “strongly agree” or “fairly often” and “very often” considered it as a barrier; and participants who chose “disagree” and “strongly disagree” or “occasionally” and “rarely” considered it as a facilitator. Questions were ranked according to the percentage of respondents who considered them as barriers or as facilitators.

Statistical analysis was performed to compare perceptions of participants from different professional groups and to examine the impact of guideline awareness on the perceptions of participants. Three questions designed only for hospital A were excluded from the analysis because they were not applicable to hospital B and 7 questions were excluded from the analysis because they were designed only for physicians and were not applicable to the other healthcare professional groups. Analysis for the remaining questions proceeded as follows. We treated questions that used a 5-point Likert scale (1 rarely, 2 occasionally, 3 sometimes, 4 fairly often and 5 very often) or (1 strongly agree, 2 agree, 3 neither agree nor disagree, 4 disagree and 5 strongly disagree), depending on whether the question was about agreement or frequency, as continuous variables [28]. Because the dependent variables examining the differences between physicians, nurses, respiratory therapists and pharmacists were not normally distributed, we conducted one-way non-parametric ANOVA. When significant differences were identified between different professional groups, post-hoc analysis was performed using the Mann-Whitney test. This test was also used to examine differences between participants who were aware of and who were not aware of the IDSA/ATS guideline for VAP management. The Mann-Whitney test works by ranking all the values from low to high. Therefore, the hypothesis tested in these tests was that means of the ranks in the two comparison groups was zero.

## Results

Surveys were distributed to 115 participants from hospital A and 85 participants in hospital B. A total of 150 participants completed the survey, among whom 87 were from hospital A and 63 participants were from hospital B. The overall response rate was 75 %. (hospital A: 75 %, hospital B: 74 %). Table 1 shows the demographics of the participants from the two hospitals.

**Table 1:** Demographics of participants

Characteristics	Hospital A (*N* = 87)	Hospital B (*N* = 63)
Gender^a^		
Male	18 (21 %)	20 (32 %)
Female	66 (76 %)	42 (67 %)
Missing	3 (3 %)	1 (2 %)
Age^a^		
Less than 25	5 (6 %)	2 (3 %)
Between 25 and 34	41 (47 %)	23 (37 %)
Between 35 and 44	19 (22 %)	14 (22 %)
Between 45 and 54	16 (18 %)	17 (27 %)
More than 55	3 (3 %)	6 (10 %)
Missing	3 (3 %)	1 (2 %)
Job position^a^		
Nurses	30 (35 %)	27 (43 %)
Pharmacists	22 (25 %)	4 (6 %)
Respiratory therapists	34 (39 %)	15 (24 %)
Physicians	1 (1 %)	17 (27 %)
Work shift ^b^		
7 am – 7 pm	40 (46 %)	36 (57 %)
7 pm – 7 am	18 (21 %)	15 (24 %)
7 am – 3 pm	19 (22 %)	13 (21 %)
3 pm – 11 pm	8 (9 %)	10 (16 %)
11 pm – 7 am	7 (8 %)	5 (8 %)
Average length working for the present employer	10.5 years	9.2 years
Average length working in the current position	8.8 years	8.8 years

^a^Total percentage does not sum to 100 % because of rounding

^b^Total percentage exceeds 100 % because more than one option could be chosen

### Barriers and facilitators

The top ranked barriers to VAP management identified in this study included (Table 2): 1) having multiple physician groups managing VAP, 2) variation in VAP management by differing ICU services, 3) physicians and level of training, and 4) renal failure complicating doses of antibiotics.

**Table 2:** List of the top-ranked barriers to VAP management

Barriers	Percent	Response category
Having multiple physician groups manage patients in the ICU complicates VAP guideline use.	67.3 %	Agree & strongly agree
There is variation in VAP management depending on what service the ICU patient is on.	64.3 %	Agree & strongly agree
ICU patients with renal failure complicate decision-making when ordering antibiotics.	57.4 %	Agree & strongly agree
Within physician service there is variation in VAP management depending on who is the VAP patient’s attending physician.	56.8 %	Agree & strongly agree
There is variation in VAP management between attending physicians and house staff in the ICU.	52.6 %	Agree & strongly agree

Facilitators to VAP management (Table 3) included presence of multidisciplinary rounds that include nurses, pharmacist and respiratory therapists, and awareness of the IDSA/ATS guideline. This awareness was associated with receiving effective training on management of VAP, keeping up to date on nosocomial infection literature, and belief that performing a bronchoscopy to diagnose VAP would help with expeditious diagnosis of VAP.

**Table 3:** List of the top-ranked facilitators to VAP management

Facilitators	Percent	Response category
Pharmacist participation on ICU rounds is beneficial.	98.60 %	Agree & strongly agree
Nurse participation on ICU rounds is beneficial.	98.00 %	Agree & strongly agree
Respiratory therapist participation on ICU rounds is beneficial.	96.70 %	Agree & strongly agree
I can readily access orders that are written for my ICU patients.	92.60 %	Agree & strongly agree
Respiratory therapy services are readily available on my ICU.	92.30 %	Fairly often & very often
Multidisciplinary management of patients occurs on my ICU.	91.90 %	Agree & strongly agree
Nurses consistently participate on ICU patient rounds.	90.30 %	Fairly often & very often
Physicians are receptive to pharmacist input in ICU patient care.	89.70 %	Agree & strongly agree
Pharmacists on my ICU effectively monitor antibiotic use.	89.30 %	Agree & strongly agree
Pharmacist participation in ICU patient management promotes appropriate antibiotic ordering.	89.00 %	Agree & strongly agree
Pharmacists consistently participate on ICU patient rounds.	88.10 %	Fairly often & very often
It is effective to have pharmacists help determine the appropriateness of ICU antibiotic de-escalation.	87.70 %	Agree & strongly agree
I can readily access the information I want on my ICU patients in the EMR.	86.90 %	Agree & strongly agree
Using VAP management guidelines helps me to manage VAP patients in the ICU.	86.70 %	Agree & strongly agree
Pharmacy intervention in antibiotic ordering leads to effective ICU VAP management.	86.30 %	Agree & strongly agree
Respiratory therapists consistently participate on ICU patient rounds.	83.20 %	Fairly often & very often
I can appropriately manage ICU patients with VAP.	83.10 %	Agree & strongly agree
VAP management guidelines interfere with my ability to manage my ICU patients.	82.30 %	Occasionally & rarely

Table 4 shows the number of participants from each professional group who were aware of and who were not aware of the IDSA/ATS guideline for VAP management. Overall, 55 % of participants indicated that they were aware of the IDSA/ATS guideline for VAP management.

**Table 4:** Proportion of participants aware of the VAP management guideline per job category

	Aware of IDSA/ATS guideline for VAP management			Total
	Yes	No	Missing	
Physicians	8	10	0	18
Nurses	30	22	5	57
Respiratory therapists	22	17	10	49
Pharmacists	23	3	0	26
Total	83	52	15	150

The next set of analyses shows results of the one-way non-parametric ANOVA and Mann-Whitney tests applied to items classified within each of nine themes identified from the content analysis of focus groups. The category with a smaller mean rank for a positively worded questions indicates that the group did better on that item than the comparison group, while a smaller mean rank on a negatively worded question indicates that the group did worse than the comparison.

None of the questions classified under three of the nine themes—*variation in practice*, *communication between providers*, and *technology and its use* differed significantly between participants who were aware of the guideline and those who were not (results not presented). For professional groups, only statistically significant (Bonferroni corrected) pairwise comparisons are presented in the paper and are summarized in Table 5 (Online supplemental material).

**Table 5:** Comparisons of professional groups’ perceptions and beliefs about various items related to VAP management

Theme	Item	Mean Rank	*P*-Value
Communication between providers	They would benefit by receiving clinical progress reports feedback on VAP patients after they are discharged from the ICU	Physicians vs. respiratory therapists, 43.1 vs 72.4	0.03
		Physicians vs. pharmacists, 43.1 vs 85.2	0.02
	Could more readily access information on ICU patients from the EMR	Respiratory therapists vs. nurses, = 62.8 vs 85.0	0.02
Difficulty in diagnosing VAP	Being able to perform a bronchoscopy in the ICU helps the physician to expeditiously diagnose VAP	Physicians aware of the guideline vs. those not aware of it, 57.4 vs 68.7	0.05
Education related to VAP and VAP management	Received effective training on VAP management	Participants aware of the guideline vs. those not aware of it, 56.7 vs 83.6	<.01
	Kept up-to-date on nosocomial infection literature	Participants aware of the guideline vs. those not aware of it, 54.73 vs 85.81	<.001
	Could appropriately manage ICU patients with VAP	Participants aware of the guideline vs. those not aware of it, 58.14 vs 71.35	0.01
	Believe that they could easily interpret quantitative culture results related to VAP *(applicable to physicians only)*	Participants aware of the guideline vs. those not aware of it, 55.92 vs 72.17	0.01
	Believe that they could accurately diagnose ICU patients with VAP *(applicable to physicians only)*	Participants aware of the guideline vs. those not aware of it, 41.81 vs. 53.16	0.03
	Kept up-to-date on nosocomial infection literature	Pharmacists vs. nurses, 58.8 vs 83.9	<.01
Guideline awareness and use	ICU VAP management order sets would facilitate VAP management	Pharmacists vs. respiratory therapists, 51.5 vs 88.5	<.01
		Physicians vs. respiratory therapists, 54.6 vs 88.5	<.01
	VAP management guidelines interfere with their ability to manage my ICU patients	Respiratory therapists vs. pharmacists, 44.0 vs 72.1	<.01
Management of the condition	Having nurses float between ICUs interferes with standardized VAP patient management	*Participants aware of the guideline vs. those not aware of it, 70.1 vs 57.5	<.001
	Physicians are receptive to respiratory therapist input in ICU patient care	Physicians vs. respiratory therapists, 56.0 vs 87.9	<.01
	Physicians are receptive to pharmacists’ input in ICU patient care	Physicians vs. respiratory therapists, 52.3 vs 79.5	0.02
	ICU patients with renal failure complicate decision-making when ordering antibiotics	Physicians vs. pharmacists, 35.6 vs 70.7	<.01
		Physicians vs. respiratory therapists, 35.6 vs 79.2	<.01
		Nurses respiratory therapists, 56.1 vs 79.2	0.02
Provider responsibilities	It is effective to have pharmacists help determine the appropriateness of ICU antibiotic de-escalation	Participants aware of the guideline vs. those not aware of it, 57.8 vs 73.2	<.001
		Pharmacists vs. respiratory therapists, 49.9 vs 82.5	<.01
	Attending physicians should be responsible for educating house staff on VAP management guidelines	Participants aware of the guideline vs. those not aware of it, 61.6 vs 74.5	0.04
	Respiratory therapy does not respond promptly to mini-BAL orders for ICU patients with suspected VAP	Participants aware of the guideline vs. those not aware of it, 51.54 vs 41.3	0.05
	Pharmacy intervention in antibiotic ordering leads to effective ICU VAP management	Pharmacists vs. respiratory therapists, 50.5 vs 86.2,	<.01
		Nurses vs. respiratory therapists, 67.8 vs 86.2,	0.04
	Multidisciplinary management of patients occurs on their ICU	Pharmacists vs. respiratory therapists, 63.8 vs 88.1	0.04
	Pharmacists on their ICU effectively monitor antibiotic use	Pharmacists vs. respiratory therapists, 50.1 vs 82.9	<.01
Technology and its use	Having an electronic medical record (EMR) reduces the time necessary to diagnose VAP in the ICU	Physicians vs. nurses, 42.6 vs 75.9	0.04
		Physicians vs. pharmacists, 42.6 vs 76.8	0.02
Use of clinically indicated tests	ICU respiratory therapists are capable of performing mini-BALs	*Participants aware of the guideline vs. those not aware of it, 63.8	0.03
	ICU respiratory therapists are capable of performing mini-BALs	Respiratory therapists vs. pharmacists, 49.3 vs 84.2	<.01
	More clinically useful specimens are collected when mini-BALs are performed	Respiratory therapists vs. physicians, 49.3 vs 91.1	<.01
Variation in practice	There is variation in VAP management depending on what service the ICU patient was on	Pharmacists vs. respiratory therapists, 47.9 vs 84.3	<.01
	There is variation in VAP management depending on who the VAP patient’s attending physician was	Pharmacists vs. respiratory therapists, 52.3 vs 79.8	<.01
	There is variation in VAP management between attending physicians and house staff in the ICU	Pharmacists vs. respiratory therapists44.2 vs 75.5	<.01
		Pharmacists vs. nurses, 44.2 vs 76.0	<.01
	Antibiotic ordering practices vary between house staff and attending physicians in the ICU	Respiratory therapists vs. pharmacists, 33.7 vs 63.2	0.02
		Respiratory therapists vs. physicians, 33.7 vs 64.3	
		Respiratory therapists vs. physicians, 33.7 vs 64.3	0.03
		Nurses vs. pharmacists, 45.0 vs 63.2	0.04

Note: Only statistically significant (Bonferroni corrected) pairwise comparisons are presented in this table

A 5-point Likert scale used was as follows:1 strongly agree, 2 agree, 3 neither agree nor disagree, 4 disagree and 5 strongly disagree or 1 rarely, 2 occasionally, 3 sometimes, 4 fairly often and 5 very often. Therefore, a professional group with a smaller rank was more likely to believe or report the stated item than the professional group with a larger mean rank. The opposite is true for items with *

*EMR* electronic medical record, *VAP* ventilator-associated pneumonia, *ICU* intensive care unit, *mini-BAL* mini-bronchoalveolar lavage

Compared to other professional groups, a higher proportion (71 %) of respiratory therapists agreed that more clinically useful specimens are collected when mini-BALs are performed (followed by physicians (61 %), nurses (45 %) and then pharmacists (25 %), (*p* = 0.01).

## Discussion

VAP accounts for a major proportion of anti-infective use in the ICU. Recent data indicate that antibiotic use is significantly higher in ICU patients compared with non-ICU patients for most antimicrobials [29] Optimizing management of VAP is clearly essential yet remains variable [30, 31] and is associated with suboptimal prescribing practices [32].

Antibiotic treatment guidelines have emerged as a potentially effective means of avoiding unnecessary antibiotic administration, increasing the effectiveness of prescribed antibiotics, and reducing antimicrobial resistance as well [33].

In our study, we attempted to understand the adoption and uptake of a national VAP diagnosis and management guideline [12]. Overall, we found that 55 % of participants indicated that they were aware of the IDSA/ATS guideline. The top ranked barriers to VAP management included: 1) having multiple physician groups managing VAP, 2) variation in VAP management by differing ICU services, 3) physicians and level of training, and 4) renal failure complicating doses of antibiotics. Changes in renal clearance following renal failure make it difficult to establish precise antimicrobial dosing [34].

One of the top barriers to VAP management, having multiple physician groups managing VAP may be more likely to lead to poor patient outcomes particularly because of the increased likelihood of communication errors in the presence of multiple providers [35, 36].

Most respondents felt that nurse, pharmacist, and respiratory therapy participation in rounds was beneficial for VAP management. This finding further emphasizes the need for multidisciplinary rounds which have been associated with improved patient outcomes such as reduced mortality [37, 38] and reduced length of hospital stay [39].

Participants who were aware of the guideline also believed that they received effective training on management and kept up to date on nosocomial infection literature. However, they were less likely to believe that ICU respiratory therapists are capable of performing mini-BALs.

Compared to nurses, more pharmacists believed that there was variation in VAP management. This is not surprising because most of the variation in VAP management occurs in antibiotic use and pharmacists have a key role in that decision-making process. Awareness of the guideline was also more likely to be associated with belief that performing a bronchoscopy to diagnose VAP would help with expeditious diagnosis of VAP.

Other studies have shown that implementing the IDSA/ATS guideline by customizing it into center-specific guidelines was associated with increased adherence to guideline diagnostic criteria for nosocomial pneumonia and guideline-concordant empiric antibiotics [40, 41]. Our findings complement these existing studies by identifying perceptions of the many different types of healthcare workers (HCWs) in ICU settings.

Our study has limitations. Our survey was limited to two institutions in Wisconsin and thus may limit generalizability of our findings. We did not measure guideline adherence or correlate it with outcomes. A recent study found that guideline-adherent initial intravenous antibiotic therapy led to better patient outcomes and was less expensive than non-guideline adherent therapy [42]. However, other studies have shown that guideline adherent care alone did not necessarily improve patient outcomes such as the frequent need for mechanical ventilation and all-cause 30 day mortality [43, 44]. We did not collect further data on the demographics of the respondents to allow comparisons between attendings and trainees for example. Knowledge of the guidelines might be different between these categories. We had less physician participation than other types of HCWs. Finally, as with any survey-based studies, we also cannot rule out the possibility of variability in question interpretation [45, 46].

These limitations notwithstanding, our findings have implications for antibiotic stewardship teams, clinicians, and organizational leaders.

## Conclusion

About half of the respondents reported that they were aware of the IDSA/ATS guideline. Awareness of guidelines was associated with a belief that respondents received effective training on management and kept up to date on nosocomial infection literature. The top ranked barriers to VAP management included having multiple physician groups managing VAP, variation in VAP management by differing ICU services, physicians and level of training and renal failure complicating doses of antibiotics. Nurse, pharmacist and respiratory therapy participation in rounds was viewed as beneficial to VAP management by most respondents. Future studies should rigorously examine the impact of guideline adherence for VAP management to clinical patient outcomes and assess process measures to gauge degree and success of guideline implementation.

## Abbreviations

ANOVA, Analysis of variance; ATS, American Thoracic Society; HCWs, healthcare workers; ICU, intensive care unit; IDSA, Infectious Diseases Society of America; mini-BAL, mini-bronchoalveolar lavage; SEIPS, Systems Engineering Initiative for Patient Safety; VAP, ventilator-associated pneumonia
